# The Burden of Malnutrition among Adults Residing in Arba Minch Health and Demographic Surveillance Site (HDSS): A WHO STEPS Survey

**DOI:** 10.1155/2020/6986830

**Published:** 2020-01-28

**Authors:** Befikadu Tariku Gutema, Adefris Chuka, Mekdes Kondale, Gistane Ayele, Mesfin Kote, Zerihun Zerdo, Behailu Merdekios, Tsegaye Tsala, Alazar Baharu, Gebresilasea Gendisha Ukke

**Affiliations:** ^1^Arba Minch University, Department of Public Health, Arba Minch, Ethiopia; ^2^Arba Minch Health and Demographic Surveillance System (HDSS), Arba Minch, Ethiopia; ^3^Save the Children International, Konso Field Office, Konso, Ethiopia; ^4^Arba Minch University, Department of Medical Laboratory, Arba Minch, Ethiopia; ^5^Arba Minch University, Department of Computer Science, Arba Minch, Ethiopia; ^6^Arba Minch University, Department of Midwifery, Arba Minch, Ethiopia

## Abstract

**Background:**

Malnutrition is one of the main underlying risk factors for the deaths due to different diseases. The aim of this study was to assess the prevalence and factors associated with underweight and overweight among adults residing in Arba Minch Health and Demographic Surveillance Site (HDSS), Southern Ethiopia.

**Methods:**

A community-based cross-sectional survey was conducted from April to June 2017. The data collection procedures and 3,368 calculated sample size were based on the World Health Organization (WHO) STEPwise approach to Surveillance guideline. Using the surveillance data of Arba Minch HDSS, simple random sampling technique was implemented to identify individuals for the study. To assess the presence of association, the multinomial logistic regression model was used.

**Results:**

The mean (SD) body mass index of the participants was 21.5 4.90 kg/m^2^. From 3,346 participants, 23.3% of the study participants were affected by malnutrition (10.8% and 12.5% were overweight and underweight, respectively). The prevalence of underweight was increased significantly among individuals aged 45–54 years and 55–64 years (adjusted odds ratio (AOR) 1.70 and 1.93, respectively) compared with those who were 25–34 years old. Belonging to households with higher wealth index quintile (2^nd^ quintile AOR is 0.58 and 4^th^ quintile AOR is 0.66) has decreased the chance of adult individual to be underweight compared with the poorest households. On the other hand, the prevalence of overweight was significantly higher among females (AOR 1.60), urban residents (AOR 1.72), those with formal education (primary AOR 1.89 and secondary and above AOR 1.94), and higher wealth index (5^th^ quintile AOR 1.87).

**Conclusion:**

One in five adult individuals was malnourished in the study area. The double burden of malnutrition at the population level is becoming a challenge for this community, as both underweight and overweight are becoming prevalent. Sex, age, residency, educational status, current tobacco use, occupation, and wealth index were identified as important determinates of under- and overweight.

## 1. Background

Malnutrition refers to deficiency, excesses or imbalance in a person's intake of energy and/or nutrients. When shortage (undernutrition) coexists with taking excess of the nutrients (overnutrition) in a population, household, or individuals, it is termed as double burden of malnutrition. Undernutrition includes underweight and micronutrient deficiencies and overnutrition is expressed in terms of overweight and obesity [1, 2]. The World Health Organization (WHO) defines underweight, overweight, and obesity as body mass index (BMI) less than 18.5 kg/m^2^, 25 kg/m^2^ to 30 kg/m^2^, and greater than 30 kg/m^2^, respectively [3].

Even if one of the targets of WHO Global action plan for the prevention and control of noncommunicable diseases is to stop the rise in obesity [4], the mean BMI has been increasing and the prevalence of obesity was increased by nearly twice after a decade (from 1998 to 2008) [5]. The trend of overweight and obesity is estimated to increase in most of the countries [6]. Health-related quality of life diminishes as the individual becomes under- and overweight. Some of the indicators of the health-related quality of life that diminishes as individuals becoming under- and overweight are physical health, mental health, activity limitation, and generated considerable health-related cost [7, 8].

Most studies reveled that change in BMI have V-shaped (goes beyond the cutoff point for under- and overweight) association with the risk of death [8–13]. Overweight and obesity are the risk factors for the death due to different chronic diseases including cardiovascular and cancer [8, 14–18]. In addition, there is an increased mortality among underweight individuals compared with normal-weight individuals [9, 10, 13]. In general, both under- and overweight are related with increased overall risk of mortality [10].

The WHO STEPwise approach to Surveillance (STEPS) focuses on obtaining core data on the established risk factors for the major chronic noncommunicable disease burden. One of the established risk factors for the development of noncommunicable disease indicator is BMI. The analysis of this article was based on the first two steps of WHO STEPS approach [19]. The objective of the study was to assess the prevalence and factors associated with malnutrition (underweight and overweight) among adult residing in Arba Minch health and demographic surveillance site (HDSS), which was collected based on STEPS approach.

## 2. Methods

### 2.1. Study Setting and Area

The study was conducted in Arba Minch HDSS, which is located in Arba Minch Zuria District, Southern Ethiopia. Arba Minch Town, administrative town of the district is located 505 km south from the capital city, Addis Ababa. Arba Minch HDSS includes nine Kebeles (the lowest administrative unit of Ethiopia) of Arba Minch Zuria District. Eight of the nine HDSS Kebeles are rural and the remaining one is semiurban. According to the 2016 report from the site, 14,754 households and 74,107 individuals (male 37,130 and female 36,977) were living in the site.

### 2.2. Study Design, Period, and Population

A community-based cross-sectional survey was conducted from April to June 2017. The source population was adult residents (25–64 years) of Arba Minch HDSS. Based on the 2016 site report, 24,800 (nearly half (52.2%) of them were females) eligible individuals were included as source population. Pregnant mothers and women who have history of recent delivery up to 8 weeks were excluded from the study.

### 2.3. Sample Size Determination and Procedures

According to WHO STEPS guideline for sample size calculation, the study population was categorized into eight groups based on the four age and sex categories. Raised blood pressure is one of the risk factors for chronic noncommunicable diseases [19], and its prevalence in southern Ethiopia was considered for the calculation of the sample size (which showed that the prevalence was 22.4%) [20]. Using a single population proportion formula and design effect of 1.5, the estimated sample size for a group was 396. With the consideration of 5% nonresponse rate and eight groups in order to have an adequate level of precision for each age-sex estimate, the final sample size was 3368. The sampling frame was extracted from Arba Minch HDSS database using sex, date of birth, and individual and household identifications as extraction variables. Based on sex and age category (25–34 years, 35–44 years, 45–54 years and 55–64 years), the sampling frame was stratified in to eight groups and simple random sampling techniques was implemented using STATA version 14 to select the study participants from each stratum.

### 2.4. Data Collection Instruments

Data collection instruments were adapted from WHO STEPS instruments. From three levels of STEPS approach, only step one and two were applied for this study. The first step is the questionnaire based, which was designed to obtain core data on sociodemographic information, tobacco and alcohol use, food intake, and physical activity. Height and weight measurements are included from second step and measured according to the WHO STEPS approach. The physical activity level was assessed using the Global Physical Activity Questionnaire, which focus on the activity at work, travel to and from places, and recreational activities of the individual [21]. All the modifications were done in accordance with the STEPS manual [19]. In addition to WHO STEPS approach, variables for food security status, wealth index and mental stress were included in the questionnaire. The Household Food Insecurity Access Scale (HFIAS) Questionnaire, which was developed and validated by Food and Nutrition Technical Assistance, was used to assess the food security status of the households [22]. Household wealth index questions were obtained from Ethiopian Demographic and Health Survey (EDHS), which was based on the household ownership of productive asset and household characteristics [23]. Mental stress was examined using Self-Reporting Questionnaire (SRQ-20), which was used for easily acquired mental health symptoms of the participants [24].

### 2.5. Data Collection Process

High school completed data collectors and diploma level supervisors participated in data collection. Training was given on the interview technique, content of the questionnaire, and measurement techniques. The data collection was done according to the WHO stepwise approach. Interview and measurement were conducted at the participants' house. Body weight (to the nearest 0.5 kg) was taken with the participant on bare feet and with light clothing using the SECA digital scale. Height (to the nearest 1 cm) was measured using a stadiometer with participants wearing no shoes and without headwear.

### 2.6. Data Quality Control

Experienced data collectors and supervisors of Arba Minch HDSS were involved in the data collection process. Training was given for three days on data collection material and measurement procedures. Pretest was conducted on 2% of the sample size. After the pretest, modifications were made on the tools based on the identified gaps. Supervisors had monitored the whole data collection process and checked the data for completeness every day during the data collection time. Standardized measuring instruments was used for physical measurements. To increase the response rate, the data collectors repeatedly visited (at least three times) those participants who were not present at the house during data collection time.

### 2.7. Ethics Approval and Consent to Participate

Ethical approval was obtained from Institutional Review Board of Arba Minch University. A formal letter was written to different administrative bodies and organizations to obtain permission to conduct the research in the settings. Verbal informed consent was obtained from study participants before interview and physical measurements. The privacy of the study participants was maintained by interviewing the participants alone.

### 2.8. Data Processing and Analysis

EPI-data version 3.1 statistical software was used for data entry, and the data were exported to STATA version 14 for data management and analysis. By using household assets variables, the wealth index was constructed via the principal component analysis method [23]. Food security status of the household was generated in accordance with the Food and Nutrition Technical Assistance's HFIAS method [22]. For the calculation of the physical activity level, the total time spent in physical activity during a typical week and the intensity of the physical activity are taken into account and categorized to three levels (high, moderate, and low physical activity levels) [21]. Heavy alcohol consumption was generated based on the individual consumption of alcohol with six or more drinks on a single occasion. Fruit and vegetable consumption level was considered per week, and flesh food consumption was considered throughout the year and categorized based on the frequency of consumption. Based on SRQ-20, mental stress was categorized in to three (mild, moderate, and severe). BMI was calculated as weight in kilograms divided by square of height in meters (kg/m^2^). For categorization, less than 18.5 kg/m^2^, 18.5–25 kg/m^2^, and over 25 kg/m^2^ were considered for underweight, normal, and overweight/obesity. To assess the presence of association between dependent and independent variables, the chi-squared test was used. Then, based on this analysis, all variable which has *P* value less than 0.20 was entered into a multinomial logistic regression model. To outline the independent predictors of the BMI categories, statistical significance at *P* value less than 0.05 was considered. Collinearity among the independent variables was assessed by measuring their variance inflation factor.

## 3. Results

A total of 3,346 adults participated in the study with response rate of 99.4%. The mean (SD) age of the study participants was 44.6 (11.2) years. Half of the study participants were women (50.0%) and farmers (53.2%). Most of the participants were married (87.9%). More than two-third (69.8%) of them did not attend formal education (Table 1). The most common ethnic background was Gamo (81.1%) and followed by Zeyise (8.7%) and Wolyita (5.5%).

The mean (SD) BMI of the participants was 21.5 (4.90) kg/m^2^ (21.4 (3.44) kg/m^2^ and 21.7 (6.01) kg/m^2^ for male and female, respectively). There was no significant (*P* value = 0.056) difference between mean BMI among sex category. From the total, 23.3% (95% CI: 21.9%–24.8%) of the participants were malnourished. The prevalence of overweight and underweight was 10.8% (95% CI: 9.8%–11.9%) and 12.5% (95% CI: 11.4%–13.7%), respectively. The prevalence of obesity was 1.9% (95% CI: 1.5%–2.4%).

About one-fifth (20.2%) of the participants were current consumers of tobacco, and one-tenth (11.5%) consumes alcohol heavily. Nearly 10% (329) of the participants did not eat meat, fish, and poultry for a year (Table 2).

Age group, sex, marital status, educational status, occupation, wealth index, and current consumption of tobacco were significantly associated with underweight in bivariate analysis. Overweight was significantly associated with age group, sex, residency, marital status, educational status, religion, occupation, wealth index, food security status, current consumption of tobacco, heavy consumption of alcohol, fruit and vegetable consumption level, and mental stress (Table 3).

Based on multinomial logistic regression analysis, the prevalence of underweight increases with increase in age, especially significant increase was observed among age group between 45 to 54 (adjusted odds ratio (AOR) 1.70, 95% CI 1.21–2.39) and 55 to 64 (AOR 1.93, 95% CI 1.37–2.74) years compared with the age group between 25 to 34 years. Regarding the occupation of the individuals, the proportion of underweight was increase nearly by half among housewives (AOR 1.56, 95% CI: 1.14–2.12) compared with farmers. In addition, the likelihood of being underweight among daily laborer, employee of organization, and unemployed was nearly two times ((AOR 2.01, 95% CI: 1.35–3.00) (AOR 2.69, 95% CI: 1.47–4.95), and (AOR 2.08, 95% CI: 1.24–3.48), respectively) compared with the farmers. There was significant decrease in prevalence of underweight among individuals with 2^nd^ quintile (AOR 0.58, 95% CI 0.42 to 0.80) and 4^th^ quintile (AOR 0.66, 95% CI 0.47 to 0.94) wealth index compared with the 1^st^ quintile wealth index. From the behavioral factors, only current consumption of tobacco was significantly associated with underweight. The probability to be underweight among current tobacco consumers was increased by over half (AOR 1.58, 95% CI: 1.18–2.13) compared with the noncurrent tobacco users (Table 3).

The prevalence of overweight was higher among females (AOR 1.60, 95% CI: 1.17–2.20) compared with male. In addition, the prevalence of overweight was higher among urban residents (AOR 1.72, 95% CI: 1.30–2.27) compared with rural residents. Most overweight individuals were among those adults with formal education (primary education (AOR 1.89, 95% CI: 1.42–2.51) and secondary and above education (AOR 1.94, 95% CI: 1.24–3.03)) compared with those who did not attend formal education. The prevalence of overweight was significantly higher among merchant (AOR 3.17, 95% CI: 2.01–5.00), housewives (AOR 1.63, 95% CI: 1.16–2.31), and employee of organization (AOR 2.05, 95% CI: 1.13–3.70) compared with farmers. Individuals from richest (5^th^) quintile had nearly 2-time (OR 1.87, 95% CI: 1.34–2.60) more likely to be overweight than individuals living in the poorest (1^st^) quintile (Table 3).

## 4. Discussion

The mean (SD) BMI among the study population was 21.5 (4.90) kg/m^2^. It is indicated that the global increase of BMI is 0.4 kg/m^2^ per decade [5]. Studies from developed and middle-income countries showed that the mean BMI was above 26 kg/m^2^ [25–27]. However, studies from different parts of Ethiopia indicated that the mean BMI is similar to the finding of this study [28–31].

In this report, the prevalence of malnutrition was 23.3% with nearly close proportion of under- and overweight. Nutritional transition, common feature of low and middle-income countries, incorporates change in the composition of the diet and physical activity level [32–34], which results in increased the prevalence of excess weight gain [35] in a society with already high prevalence of underweight. In many low- and middle-income countries, the rate of underweight is declining, and there is a dramatic increase in overweight and associated noncommunicable diseases [28, 36]. As indicated in this result also, there is an increase of double burden of malnutrition in the community, with nearly similar proportion of under- and overweight among adults [1].

The prevalence of overweight was 10.8%. Studies from developed countries showed that the prevalence of overweight was higher than the finding of this report. For instance, some studies showed that nearly 35% of American, 50% of European, and 50% of Iranian were overweight [25–27]. In addition, the global prevalence of overweight was around 38% based a report on global prevalence of overweight. However, the levels of the overweight and obesity in developing countries were much lower than those in developed countries [37]. A meta-analysis on the prevalence of obesity among west African population indicated that it was around 10% in the period of 2000 and 2004 [38]. A report based on systematic analysis indicated that the prevalence of overweight for Ethiopian in 2013 was nearly 9% [37]. Similarly, a report based on EDHS 2011 indicated that the prevalence of overweight among women was 8% [31]. But, the prevalence of overweight and obesity among Gondar town population with age greater than 35 years was 25.3% and 5.6%, respectively [39]. In general, the prevalence of overweight of this study population was significantly lower than that of studies conducted in developed countries. In addition, a study at Gondar town, Northern Ethiopia, showed significantly higher prevalence of overweight compared with this finding. This might be related with the study at Gondar town was on urban population and the minimum age was 35 years [39]. However, most of the reports from Ethiopia and other developing countries showed a similar finding with this study.

In this study, the prevalence of underweight was 12.5%. A report of the European Social Survey showed that the proportion of underweight was 2% [25], which was by far less than this report. According to the report of a pooled analysis of 200 countries, Asia and sub-Saharan Africa countries were the area with the largest number of people with underweight lived in 2014. It also described that the prevalence of underweight in East Africa was nearly 12% and a total of 9.2 million adult population was underweight in Ethiopia [28]. Similar to the proportion of this study, a study conducted East and West Gojjam indicated that 12.6% women were underweight [30]. But, a report based on the data of the EDHS 2016 indicated that the prevalence of underweight among women was 22.4% [23], which was significantly higher than this report. Even if there were discrepancy among studies conducted in Ethiopia on the prevalence of underweight, generally the prevalence was significantly higher compared with the finding of different countries.

The finding of this study indicated that there was no significant difference on the prevalence of underweight between male and female participants. A pooled analysis to show trends in adult BMI showed that more men were underweight than women in east Africa in 2014 [28]. Regarding overweight, the prevalence was significantly higher among females as compared with males. A meta-analysis on the adults of West African population and a cross-sectional report from studies conducted in America and European countries showed that there was also significantly higher prevalence of overweight among females [25, 26, 38].

In this study, the prevalence of underweight was significantly higher among the older age group but there was no significant difference. A study conducted in America showed that the age has V-shaped relationships with self-reported BMI. Even if there was no significant difference among age groups regarding overweight, the study indicated that underweight increases with age [7]. Contrarily, different reports based from European society, among Iranian and a study at Jimma, Ethiopia, indicated that older adults were significantly more overweight and obese than middle age and younger adults [25, 27, 29, 40]. This difference between the finding of this study and other might be because most (83.7%) of the population of this study were rural residency.

This finding showed that the prevalence of overweight increase among urban population compared with the rural. It is similar to the study conducted on Iranians [27] and result of a meta-analysis on adult West African populations which showed that urban residents had particularly high risk of overweight/obesity [38]. But, a study conducted in three European countries showed that the prevalence of obesity had no significant difference among the place of residency [40].

The finding indicated that the prevalence of overweight was increased among individuals with formal education. However, the prevalence of underweight was not associated with educational status of the population. A report based on Bangladesh Demographic and Health Survey 2004 indicated a similar finding regarding the overweight and educational status among women of the reproductive age group [41]. In addition, pooled analyses of 20 prospective cohorts study in Asia reported that higher BMI is related to higher educational status [12]. In contrary, a study in three European countries indicated that obesity was more prevalent among the less educated individuals, especially among less educated women [40]. A systematic review on educational attainment and obesity indicated the relationship between obesity and educational attainment depends on country's level of development. For developed country, the relationship is inverse, whereas for developing country, it is a positive association [42].

According to the finding of this study, the prevalence of underweight was higher among current tobacco users. Similarly, a study among adult population in the United States showed that there was an inverse relationship between current smoking and self-reported BMI [7]. A comparative study between smokers and nonsmokers in Canadians showed that smokers consumed less healthy diet [43], which might be a reason for increased prevalence of underweight among this group.

This report indicated that the prevalence of underweight reduced and overweight increased at higher wealth index quintiles. Similarly, a repeated cross-sectional analysis in India indicated that high socioeconomic status women were more likely to be overweight, and low socioeconomic status were becoming underweight [44]. In addition, a study in Jimma town, Ethiopia, showed similar trend with the finding of this report related with the wealth index. The report indicated that the BMI increases with increase in the income of the household [29]. In contrast, data from European Social Survey showed that household with lower income had a higher probability to become obese than the higher income household [25]. The wealth index for the developing countries showed similar pattern on the BMI. But, the finding is not consistent with that of the European study, which indicate opposite direction especially for the prevalence of obesity and overweight with the economic status of the household. This might be due to that fact that wealth enhances the individual's accessibility to food and reduced energy expenditure in developing countries. In developing countries, high-income groups benefit from a more dynamic marketplace and lower-income group convergent to low-quality diet [45], which increase riskiness of lower income households for underweight and high-income households for overweight.

### 4.1. Strength and Limitations of the Study

Consumption-related questions (alcohol and food) are heavily affected by recall bias, which is one of the limitations of the study. HFIAS was used to assess one dimension of food insecurity status and only assess the monthly status of the household. Even if it is sensitive, mental stress was assessed by self-reported question. Being community-based, use of simple random sampling technique and large sample size of this study makes it representative of adult population of the community. In addition, we used recently updated (less than 6 month) sampling frame of Arba Minch HDSS, which increased its response rate. To assure validity of findings, the study has used a modified version of the standardized methods recommended by WHO STEPS guideline for chronic noncommunicable disease surveillance.

## 5. Conclusion

One in five adult individuals was malnourished in the study area with nearly similar proportion among under- and overweight. More than one in ten adults were underweight, which indicate that it is still high in this population. The double burden of malnutrition at the population level is becoming a challenge for this community, as both underweight and overweight are becoming prevalent. The prevalence of underweight was increased among higher age groups, current tobacco consumption, daily laborer, housewives, employed at organization, unemployed individuals, and lower wealth index of their households. The prevalence of over-weight was higher among females, urban residents, those with formal education, merchants, housewives, employee of an organization, and higher wealth index households. Intervention focus on reduction of adult underweight should give due attention to older adults, daily laborer, housewives, and those employed at an organization and unemployed individuals. In addition to chronic disease exposure of tobacco, its cessation intervention might have a positive impact on reducing the prevalence of underweight. Intervention for reducing the impact of overweight should give due attention to urban residents, female, educated society, and higher economic status. In general, sex, age, residency, educational status, current tobacco use, occupation, and wealth index are identified as important determinates of under- and overweight, and hence they should be considered in interventional strategies to reduce the prevalence of extreme BMIs among adult populations. In addition, future research should address the impact of educational attainment on overweight, thus supporting the education policy as a tool for overweight prevention.

## Figures and Tables

**Table 1:** Sociodemographic characteristics of the study participants with different BMI categories, Arba Minch HDSS site, Southern Ethiopia.

Characteristics	Categories	Underweight	Normal weight	Overweight
		*N* (%)	*N* (%)	*N* (%)
Sex	Male	185 (11.1)	1353 (80.8)	136 (8.1)
	Female	234 (14)	1213 (72.5)	225 (13.5)

Age group	25–34	73 (8.5)	658 (77)	123 (14.4)
	35–44	91 (10.4)	688 (78.4)	99 (11.3)
	45–54	120 (14.8)	611 (75.2)	82 (10.1)
	55–64	135 (16.9)	609 (76)	57 (7.1)

Residency	Rural	368 (13.1)	2191 (78.2)	242 (8.6)
	Urban	51 (9.4)	375 (68.8)	119 (21.8)

Marital status	Single	13 (11.2)	82 (70.7)	21 (18.1)
	Married	350 (11.9)	2288 (77.8)	303 (10.3)
	Divorced	3 (17.6)	12 (70.6)	2 (11.8)
	Widowed	44 (18.8)	162 (69.2)	28 (12)
	Separated	9 (23.7)	22 (57.9)	7 (18.4)

Educational status	No formal education	314 (13.5)	1841 (78.9)	179 (7.7)
	Primary school	87 (11.3)	547 (71.2)	134 (17.4)
	Secondary and above	18 (7.4)	178 (73)	48 (19.7)

Religion	Protestant	238 (11.3)	1603 (76.2)	263 (12.5)
	Orthodox	150 (14.2)	818 (77.6)	86 (8.2)
	Other	31 (16.5)	145 (77.1)	12 (6.4)

Occupation	Farmer	187 (10.5)	1473 (82.8)	119 (6.7)
	Daily laborer	42 (16.3)	189 (73.5)	26 (10.1)
	Merchant	12 (8.2)	92 (63)	42 (28.8)
	House wife	134 (14.7)	639 (69.9)	141 (15.4)
	Employed at Org	17 (16)	69 (65.1)	20 (18.9)
	Unemployed	24 (20.2)	84 (70.6)	11 (9.2)
	Other	3 (12)	20 (80)	2 (8)

Wealth index	The poorest	161 (15.5)	758 (73)	120 (11.5)
	2^nd^ quintile	73 (10.6)	573 (83)	44 (6.4)
	Middle quintile	72 (11.8)	473 (77.8)	63 (10.4)
	4^th^ quintile	56 (11.7)	387 (80.8)	36 (7.5)
	The richest	57 (10.8)	375 (70.8)	98 (18.5)

Food secure	Food secure	339 (12.4)	2121 (77.6)	274 (10)
	Food insecure	80 (13.1)	445 (72.7)	87 (14.2)

**Table 2:** Lifestyle characteristics of the of the study participants with different BMI categories, Arba Minch HDSS sit, Southern, Ethiopia.

Characteristics	Categories	Under weight	Normal weight	Over weight
		*N* (%)	*N* (%)	*N* (%)
Current consumption of tobacco	No	305 (11.4)	2045 (76.6)	319 (12)
	Yes	114 (16.8)	521 (77)	42 (6.2)

Heavy alcohol consumption	No	372 (12.6)	2259 (76.3)	331 (11.2)
	Yes	47 (12.2)	307 (79.9)	30 (7.8)

Fruit & vegetable consumption level	≥5 serving	262 (12.6)	1622 (78.2)	190 (9.2)
	<5 serving	157 (12.3)	944 (74.2)	171 (13.4)

Flesh food consumption	1–4 times per week	15 (11.7)	97 (75.8)	16 (12.5)
	1–3 times per month	33 (7.3)	348 (76.5)	74 (16.3)
	1–4 times per year	329 (13.5)	1886 (77.5)	219 (9)
	Never per year	42 (12.8)	235 (71.4)	52 (15.8)

Physical activity level	Low	99 (12.5)	602 (76.1)	90 (11.4)
	Moderate	48 (11.9)	302 (74.6)	55 (13.6)
	High	272 (12.7)	1662 (77.3)	216 (10)

Mental stress	Mild	273 (12.3)	1692 (76.3)	254 (11.4)
	Moderate	132 (13.3)	775 (77.9)	88 (8.8)
	Severe	14 (10.6)	99 (75)	19 (14.4)

**Table 3:** Crude and adjusted odds ratio for the association of sociodemographic and life style factors stratified by body mass index with normal weight as reference category.

Variables	Categories	Underweight			Overweight		
		COR	AOR	95% CI	COR	AOR	95% CI
Sex	Male						
	Female	1.41^*∗*^	1.24	0.94–1.62	1.85^*∗*^	1.60^*∗*^	1.17–2.2

Age group	25–34						
	35–44	1.10	1.20	0.84–1.70	0.77	1.06	0.77–1.45
	45–54	1.65^*∗*^	1.70^*∗*^	1.21–2.39	0.68^*∗∗*^	1.10	0.78–1.55
	55–64	2.01^*∗*^	1.93^*∗*^	1.37–2.74	0.49^*∗*^	0.86	0.58–1.26

Residency	Rural						
	Urban	0.81	0.77	0.55–1.09	2.87^*∗*^	1.72^*∗*^	1.3–2.27

Marital status	Unmarried						
	Married	0.62^*∗*^	0.80	0.58–1.10	0.63^*∗*^	0.72	0.51–1.02

Educational status	No formal education						
	Primary school	0.93	1.17	0.87–1.58	2.52^*∗*^	1.89^*∗*^	1.42–2.51
	Secondary and above	0.59^*∗∗*^	0.69	0.39–1.25	2.77^*∗*^	1.94^*∗*^	1.24–3.03

Religion	Protestant						
	Orthodox	1.24	1.09	0.83–1.42	0.64^*∗*^	0.83	0.61–1.12
	Other	1.44	1.10	0.69–1.76	0.50^*∗∗*^	0.82	0.42–1.57

Occupation	Farmer						
	Daily laborer	1.75^*∗*^	2.01^*∗*^	1.35–3.00	1.70^*∗∗*^	1.29	0.79–2.09
	Merchant	1.03	1.20	0.63–2.30	5.65^*∗*^	3.17^*∗*^	2.01–5.00
	House wife	1.65^*∗*^	1.56^*∗*^	1.14–2.12	2.73^*∗*^	1.63^*∗*^	1.16–2.31
	Employed at org.	1.94^*∗∗*^	2.69^*∗*^	1.47–4.95	3.59^*∗*^	2.05^*∗∗*^	1.13–3.70
	Unemployed	2.25^*∗*^	2.08^*∗*^	1.24–3.48	1.62	1.10	0.54–2.21
	Other	1.18	1.92	0.52–7.14	1.24	0.48	0.10–2.20

Wealth index	The poorest						
	2^nd^ quintile	0.60^*∗*^	0.58^*∗*^	0.42–0.80	0.49^*∗*^	0.73	0.50–1.07
	Middle quintile	0.72^*∗∗*^	0.73	0.54–1.01	0.84	1.08	0.76–1.53
	4^th^ quintile	0.68^*∗∗*^	0.66^*∗∗*^	0.47–0.94	0.59^*∗*^	0.83	0.55–1.27
	The richest	0.72^*∗∗*^	0.78	0.55–1.11	1.65^*∗*^	1.87^*∗*^	1.34–2.60

Food secure	Food secure						
	Food insecure	1.12	1.15	0.87–1.52	1.51^*∗*^	1.22	0.92–1.62

Current consumption of tobacco	No						
	Yes	1.47^*∗*^	1.58^*∗*^	1.18–2.13	0.52^*∗*^	1.01	0.69–1.48

Heavy alcohol consumption	No						
	Yes	0.93	0.92	0.64–1.31	0.67^*∗∗*^	1.02	0.65–1.6

Fruit & vegetable consumption level	≥5 serving						
	<5 serving	1.03	1.05	0.84–1.32	1.55^*∗*^	1.15	0.90–1.46

Flesh food consumption	1–4 times per week						
	1–3 times per month	0.61	0.56	0.29–1.1	1.29	1.03	0.56–1.90
	1–4 times per year	1.13	1.01	0.57–1.79	0.70	0.85	0.48–1.52
	Never per year	1.16	1.04	0.54–1.99	1.34	1.03	0.54–1.96

Mental stress	Mild						
	Moderate	1.06	1.08	0.86–1.36	0.76^*∗∗*^	0.82	0.63–1.08
	Severe	0.88	0.83	0.46–1.50	1.28	1.24	0.73–2.12

^*∗*^ *P* value <0.01. ^*∗∗*^*P* value <0.05.

## Data Availability

Raw data were obtained from the study conducted by Arba Minch Health and Demographic Surveillance Site using the WHO STEPS survey. Derived data supporting the findings of this study are available from the corresponding author upon request.

## Abbreviations

BMI: Body mass index
EDHS: Ethiopian Demographic and Health Survey
HDSS: Health and Demographic Surveillance Site
HFIAS: Household Food Insecurity Access Scale
STEPS: STEPwise approach to surveillance
WHO: World Health Organization.
